# Exploring Parental Hesitancy and Acceptance of HPV Vaccination in a Saudi Population

**DOI:** 10.3390/vaccines14030229

**Published:** 2026-02-28

**Authors:** Arwa Khaled, Khalid Orayj, Hend Talkhan, Retaj Ali, Altaf Alfifi, Shahad Bin Ghamia, Naglaa Bazan

**Affiliations:** 1 Department of Clinical Pharmacy, College of Pharmacy, King Khalid University, Abha 61413, Saudi Arabia; korayg@kku.edu.sa (K.O.); htalkhan@kku.edu.sa (H.T.); naglaabazan@cu.edu.eg (N.B.); 2 College of Pharmacy, King Khalid University, Abha 61413, Saudi Arabia; 3 Critical Care Medicine Department, Cairo University Hospitals, Cairo University, Cairo 12613, Egypt

**Keywords:** human papilloma virus, vaccine hesitancy, WHO vaccine hesitancy scale, cervical cancer, parents, Saudi Arabia

## Abstract

Background: Human papillomavirus (HPV) vaccination is a major concern and highly effective strategy for preventing cervical cancer and other related diseases. Despite the inclusion of the HPV vaccine in the Saudi national immunization program and recent school-based initiatives, vaccine uptake remains suboptimal, mainly due to the hesitancy of parents. Evidence regarding HPV vaccine hesitancy in the Aseer region of Saudi Arabia is limited. Objectives: This study aimed to assess the level of the HPV vaccine hesitancy, knowledge, attitude and barriers among parents in the Aseer region of Saudi Arabia using the World Health Organization (WHO) HPV Vaccine Hesitancy Scale (VAS). Methods: A cross-sectional study was conducted among parents residing in the Aseer region, Saudi Arabia. Data were collected using a structured questionnaire that included sociodemographic characteristics, knowledge, barriers and the validated nine-item WHO HPV Vaccine Hesitancy Scale. Data was expressed in numbers (%) for categorical variables and mean ± SD for continuous variables. Independent *t*-tests and one-way ANOVA were used for inferential statistics. Results: A total of 379 parents participated in the study. Overall, 49% of parents exhibited high HPV vaccine hesitancy. The most frequently reported barriers were safety concerns (82.6%), insufficient information (80.3%) and fear of side effects (79.4). Lower hesitancy scores were observed among parents aged ≥ 46 years than among those aged 18–25 years (*p* = 0.022), and respondents with postgraduate education were less hesitant than those with a high school education or less (*p* = 0.030). Parents whose children were fully vaccinated exhibited significantly lower hesitancy scores compared with those whose children were unvaccinated (*p* = 0.004). Conclusions: The HPV vaccine hesitancy among parents in the Aseer region is greatly influenced by the age of the parents, their educational level, and the child’s vaccination history. Higher hesitancy among younger parents, those with lower educational levels, and parents of unvaccinated children highlights priority groups for targeted interventions. Strengthening healthcare-provider recommendations and implementing culturally tailored, evidence-based communication strategies may improve HPV vaccine acceptance and support national cervical cancer prevention efforts in Saudi Arabia.

## 1. Introduction

Cervical cancer poses a significant public health threat worldwide, with over 600,000 new cases and 340,000 deaths in 2022 [1,2]. The World Health Organization (WHO) estimates that more than 95% of cervical cancer cases result from persistent, sexually transmitted high-risk human papillomavirus (HPV) infections, including oncogenic types 16 and 18 [3,4,5]. Preventing cervical cancer is highly feasible through HPV vaccination programs, routine screening (Pap/HPV tests), and appropriate follow-up treatment [6,7]. Therefore, the WHO introduced the 2020 Global Strategy for Cervical Cancer Elimination, with the goal of achieving an incidence rate below 4 per 100,000 women in all countries by 2030 [8]. The strategy has three main targets: 90% of girls fully vaccinated against HPV by age 15 before becoming sexually active; 70% of women screened by age 35 and again by age 45; and 90% of women with precancer or invasive cancer receive treatment.

Emerging evidence indicates that HPV vaccination is highly effective and generally safe, with few side effects. A 2018 Cochrane systematic review of 26 randomized controlled trials involving 73,428 participants assessed the efficacy and safety of prophylactic HPV vaccines in adolescent girls and women [9]. The review authors found that HPV vaccines protect against cervical cancer in those vaccinated between 15 and 26 years old. Additionally, protection was greater when vaccination was given before sexual debut and HPV exposure. A more recent systematic review and meta-analysis examining the impact of HPV vaccination showed a significant reduction in HPV 16 and 18 infections, anogenital wart diagnoses, and cervical intraepithelial neoplasia lesions [10]. However, global HPV vaccination coverage remains below the WHO target of 90% [11], mainly due to vaccine hesitancy among parents who are the primary decision-makers for adolescent vaccination [12,13].

The WHO’s Strategic Advisory Group of Experts (SAGE) on Immunization defines “vaccine hesitancy” as “a delay in acceptance or refusal of vaccines despite the availability of vaccination services” [14]. It is recognized as a multidimensional behavioral phenomenon influenced by various factors that differ by time, location, and vaccine type. A number of reviews of the existing literature across different countries highlight that vaccine acceptance varies globally and that no single set of factors explains vaccine hesitancy in all contexts [15,16,17]. Considering this complexity, employing evidence-based models and reliable tools to address vaccine hesitancy is essential for understanding how parents make decisions.

The HPV Vaccine Hesitancy Scale (VHS), developed in 2015 by the WHO’s SAGE on immunization, is a validated instrument for assessing vaccine hesitancy, informed by prior research and the Working Group’s expertise [14]. The scale aims to evaluate key factors that influence the decision to accept, delay, or reject vaccines, categorized into three areas: contextual (e.g., environmental or political influences), individual and group (e.g., personal or social perceptions), and vaccine-specific factors directly related to the vaccine itself [15]. Utilizing such instruments is vital for identifying modifiable factors contributing to hesitancy and for developing targeted, context-specific strategies to address vaccination concerns [18,19].

In Saudi Arabia, HPV vaccination is part of the national immunization program, and the Ministry of Health has recently launched a nationwide, free, school-based vaccination campaign [20]. Despite these efforts, HPV vaccination coverage remains low, and hesitancy persists across different regions of Saudi Arabia, posing a challenge to immunization initiatives. A 2022 cross-sectional survey involving 645 participants in the eastern region assessed knowledge and awareness of the HPV vaccine among the general population. The findings showed that only 4% of the participants had received the vaccine [21]. A more recent nationwide survey of 667 participants revealed that about one-third were hesitant to vaccinate their children against HPV, with higher hesitancy levels among families in the southern region [22]. Frequently cited barriers include fears about side effects, limited information, and the misconception that the vaccine is unnecessary unless symptoms are present [23,24,25,26]. Although there is increasing evidence at the national level, research on the HPV vaccine remains limited in the Aseer region. To address this gap, this study aims to assess HPV vaccine hesitancy, awareness, and attitudes among parents in the Aseer region of Saudi Arabia, using the HPV VHS.

## 2. Methods

### 2.1. Study Design, Reporting Guidelines, and Setting

This cross-sectional study was conducted between May and August 2025 among parents residing in the Aseer region of Saudi Arabia. The study aimed to evaluate parental knowledge, attitudes, and hesitancy regarding the human papillomavirus (HPV) vaccine.

The study was designed and reported in accordance with the Strengthening the Reporting of Observational Studies in Epidemiology (STROBE) guidelines for cross-sectional studies. All relevant STROBE checklist items were addressed during study design, data collection, analysis, and reporting.

The Aseer region is located in southwestern Saudi Arabia and is characterized by a predominantly mountainous geography, a mix of urban and rural populations, and several medium-sized cities, including Abha and Khamis Mushait. Compared with major metropolitan regions such as Riyadh and Jeddah, Aseer includes a higher proportion of semi-urban and rural communities and exhibits distinct cultural and socioeconomic characteristics. These contextual factors may influence healthcare access, preventive health behaviors, and vaccine awareness, thereby justifying a region-specific assessment.

### 2.2. Study Population and Eligibility Criteria

The study population comprised adults aged 18 years or older who were residents of the Aseer region at the time of data collection and who were parents or legal parents of at least one child. Being a parent or legal caregiver was a mandatory inclusion criterion and was verified through a screening question at the beginning of the survey. Respondents who indicated that they were not parents or legal parents were not permitted to proceed with the questionnaire.

Individuals were excluded if they were younger than 18 years, resided outside the Aseer region, declined to provide electronic informed consent, or submitted incomplete questionnaires. Only fully completed and eligible responses were included in the final analysis.

### 2.3. Sample Size and Sampling Strategy

A non-probability convenience sampling technique was employed. The sample size was calculated using the Raosoft sample size calculator (http://www.raosoft.com/sam-plesize.html, access on 26 November 2025), based on the total population of the Aseer region, with a 5% margin of error and a 95% confidence level. The minimum required sample size was 378 participants.

#### 2.3.1. Recruitment Procedure

The questionnaire link was disseminated electronically through WhatsApp community groups, the X (formerly Twitter) platform, and email distribution lists targeting parent networks within the region. The survey was open-access; however, eligibility was controlled through an initial screening question that restricted participation to parents or legal parents residing in Aseer.

To minimize duplicate responses, the survey platform restricted multiple submissions from the same device or browser session. Additionally, responses were screened during data cleaning for potential duplication based on identical demographic characteristics and submission timestamps.

Therefore, the response rate was calculated using the number of completed eligible questionnaires relative to the number of eligible respondents who accessed and initiated the survey. A total of 389 eligible participants completed the questionnaire and were included in the final analysis. 

Because the survey link was distributed through open social media platforms, the exact number of individuals who received or viewed the link could not be determined. Therefore, the response rate reflects completion among eligible respondents who initiated the survey rather than the broader regional population.

#### 2.3.2. Selection Bias and Generalizability

Given the online convenience sampling method, selection bias is possible. Individuals with internet access, active social media engagement, and higher educational or health awareness levels may have been overrepresented. Consequently, the findings should be generalized cautiously and primarily to populations with similar sociodemographic and digital access characteristics rather than to all parents in the Aseer region.

### 2.4. Data Collection Instrument and Cultural Adaptation

Data were collected using a structured, self-administered online questionnaire developed using Microsoft Forms. The questionnaire was initially prepared in English and subsequently translated into Arabic following a structured forward–backward translation process. Forward translation into Arabic was performed by a bilingual healthcare professional, and backward translation into English was independently conducted by another bilingual translator. The two English versions were compared to ensure conceptual equivalence rather than literal translation.

The Arabic version was reviewed by five academic consultants to assess clarity, content validity, and cultural appropriateness. Minor linguistic modifications were made to enhance clarity and cultural relevance. A pilot study was conducted to test comprehension and functionality, and pilot responses were excluded from the final analysis.

### 2.5. Questionnaire Structure

#### 2.5.1. Sociodemographic Characteristics and HPV Knowledge

The first section of the questionnaire collected sociodemographic data, including age, gender, relationship to the child, and vaccination history. It also assessed knowledge regarding HPV infection and HPV vaccination. Knowledge items (K1–K6) were evaluated using three response options: “Sure,” “Unsure,” and “I do not know.”

#### 2.5.2. Barriers to HPV Vaccination

The second section assessed potential informational, perceptual, and contextual barriers that might influence parental decisions regarding HPV vaccine administration. This section aimed to identify factors associated with vaccine uptake and hesitancy within the regional context.

#### 2.5.3. HPV Vaccine Hesitancy Assessment

The third section measured vaccine hesitancy using the nine-item World Health Organization (WHO) Vaccine Hesitancy Scale. Items H1–H7 assessed confidence in vaccine safety and effectiveness, whereas items H8–H9 evaluated perceived risks related to side effects and vaccine novelty. Responses were recorded on a five-point Likert scale ranging from 1 (strongly disagree) to 5 (strongly agree).

Total hesitancy scores ranged from 0 to 45, with higher scores indicating greater vaccine hesitancy. Two subscales were derived: a confidence subscale (reverse-coded H1–H7) and a perceived risk subscale (H8–H9) (World Health Organ. 2020) [8]. Internal consistency reliability was assessed using Cronbach’s alpha. The overall hesitancy scale demonstrated good reliability (α = 0.80). Reliability coefficients were also calculated separately for the confidence and perceived risk subscales.

Because a proportion of participants reported that they had never heard of the HPV vaccine prior to the survey, additional analyses were performed to address the conceptual issue that vaccine hesitancy presupposes awareness. In addition to the primary analysis including all eligible participants, a sensitivity analysis excluding respondents without prior awareness of the HPV vaccine was conducted to evaluate the robustness of hesitancy estimates. For analytical purposes, participants were categorized into two groups using the median score as the cut-off point. Participants with scores ≤ 33 were classified as having low hesitancy, whereas those with scores > 33 were classified as having high hesitancy [27].

Interpretation of mean item and subscale scores considered potential attitudinal ambivalence, particularly in cases where general pro-vaccine attitudes coexisted with concerns about vaccine safety or novelty.

### 2.6. Outcomes

The primary outcomes of the study were parental knowledge regarding HPV infection and HPV vaccination, factors associated with HPV vaccine uptake, and the level of HPV vaccine hesitancy among parents in the Aseer region.

### 2.7. Ethical Approval and Consent

The study was conducted in accordance with the principles of the Declaration of Helsinki. Ethical approval was obtained from the Ethics Committee of King Khalid University (ECM#2025-406, dated [16 February 2025]). Electronic informed consent was obtained from all participants prior to survey initiation. Participation was voluntary, and anonymity was ensured. No personal identifiers, including names, identification numbers, or IP addresses, were collected.

### 2.8. Statistical Analysis

Survey data were exported to Microsoft Excel for cleaning and coding and subsequently analyzed using the Statistical Package for the Social Sciences (SPSS), version 30. Categorical variables were summarized as frequencies and percentages, while continuous variables were presented as mean ± standard deviation. Normality assumptions were assessed prior to applying parametric tests.

Independent *t*-tests and one-way analysis of variance (ANOVA) were used to examine associations between demographic variables and vaccine hesitancy scores. Where statistically significant differences were observed in ANOVA, appropriate post hoc comparisons were performed. A *p*-value of less than 0.05 was considered statistically significant.

Multiple linear regression analysis was conducted to examine factors independently associated with parental HPV vaccine hesitancy score, which was treated as a continuous outcome variable. Demographic, knowledge-related, and attitudinal variables were entered simultaneously into the model, and statistical significance was set at *p* < 0.05.

## 3. Results

A total of 379 parents participated in the study (mean age = 34.2 years, SD = 8.5). Most participants were young to middle-aged adults, with the largest proportion in the 26–35-year age group, with a slightly higher representation of females than males.

More than half of the participants had completed a diploma or bachelor’s degree, while smaller proportions reported either high school education or less or postgraduate education. The majority of parents reported that they had previously heard about the vaccine. Detailed sociodemographic characteristics are presented in Table 1.

Overall, knowledge levels were modest and characterized by substantial uncertainty across most items. The greatest gaps were observed in awareness of HPV prevalence and the recommended age for vaccination, where a large proportion of parents reported being unsure. Knowledge of the association between HPV and certain cancers and of gender-inclusive vaccination recommendations was moderate but remained incomplete.

Despite these knowledge limitations, parents generally expressed more favorable perceptions regarding the importance and safety of the HPV vaccine, although uncertainty about vaccine safety remained considerable. These findings indicate that insufficient knowledge—particularly regarding disease burden and vaccination timing—may represent important barriers to informed decision-making among parents (Table 2). 

Concerns related to vaccine safety and insufficient information were the most frequently identified barriers. A large majority of parents reported safety concerns, lack of adequate information, and fear of side effects as key reasons for hesitancy.

Approximately half of the parents identified religious or cultural beliefs and the perception that their child was too young for vaccination as important barriers. In contrast, cost-related concerns and the perception that the child was not at risk for HPV infection were reported less frequently.

These findings suggest that informational and safety-related concerns represent the dominant barriers to HPV vaccination among parents, whereas structural and financial factors appear to play a comparatively smaller role in this population (Table 3).

The analysis of parental attitudes toward and beliefs about the HPV vaccine revealed predominantly positive attitudes with concurrent safety concerns. All scale items demonstrated mean scores above the midpoint (3), ranging from 3.53 to 3.79, indicating overall favorable attitudes toward HPV vaccination. Parents reported particularly strong agreement with statements related to the protective value of the vaccine for their child’s health, its benefits for individual and community health, and their willingness to follow healthcare provider recommendations. At the same time, safety-related concerns remained evident, as reflected in relatively high agreement with statements expressing worry about potential side effects and uncertainty regarding long-term safety. Trust in the information received about the HPV vaccine showed comparatively lower scores than other positive attitude items, indicating a potential area for improvement. Overall, the findings suggest that positive attitudes toward HPV vaccination coexist with notable safety concerns among parents, as shown in Table 4.

Participants’ total hesitancy scores ranged from 0 to 45. The average mean was 32.35 (SD = 8.14), with recorded scores covering the full scale (Min = 0, Max = 45) and median = 33. The participants’ scores were divided into two categories: 194 (51.2%) participants showed a low hesitancy score (≤33), and 185 (48.8%) showed a high hesitancy score (≥33) (Table 5).

Statistically significant differences in HPV vaccine hesitancy scores were observed with respect to caregiver age, education level, and vaccination history. Parents aged 46 and older reported significantly lower hesitancy than those aged 18–25 (*p* = 0.022), and respondents holding postgraduate degrees were less hesitant than those whose highest education was high school or less (*p* = 0.03). Moreover, parents of fully vaccinated children scored markedly lower than their unvaccinated counterparts (*p* = 0.004). In addition, hesitancy scores differed significantly according to willingness to vaccinate the child if recommended by a trusted physician (*p* < 0.001).

In contrast, child gender (*p* = 0.594), caregiver relationship to the child (mother vs. father vs. other guardian) (*p* = 0.121), parent gender (*p* = 0.571), and prior vaccine awareness (*p* = 0.238) did not differ significantly in mean hesitancy scores. (Table 6).

Several demographic and attitudinal factors were significantly associated with hesitancy after adjustment for covariates. Parent age was inversely associated with hesitancy, indicating that older parents tended to report lower hesitancy scores. In contrast, higher educational attainment was associated with slightly higher hesitancy. Relationship to the child was also significantly associated with hesitancy, whereas child gender showed a marginal but non-significant association.

Among attitudinal factors, perceiving HPV vaccination as important for one’s child was associated with lower hesitancy. The strongest association in the model was observed for parents’ reported likelihood of vaccinating their child if recommended by a trusted physician, which was linked to substantially lower hesitancy scores.

In contrast, knowledge-related variables and prior exposure to HPV vaccine information were not significantly associated with hesitancy after adjustment for demographic and attitudinal factors. These findings suggest that attitudinal and trust-related factors may play a more prominent role in HPV vaccine hesitancy than knowledge alone in this sample. Variance inflation factor values indicated no evidence of problematic multicollinearity (Table 7).

## 4. Discussion

HPV vaccination is a key strategy for the primary prevention of cervical cancer worldwide; however, vaccine hesitancy remains a major barrier to optimal uptake across many countries. In addition to cervical cancer, persistent HPV infection is implicated in several other malignancies, including anal, oropharyngeal, penile, vulvar, and vaginal cancers, further emphasizing the broader public health importance of HPV vaccination. Globally and within Arab countries, parental concerns related to vaccine safety, limited awareness, and sociocultural factors have been consistently identified as key influences on HPV vaccine acceptance [28,29,30]. Despite the inclusion of HPV vaccination in the Saudi national immunization program, parental acceptance remains suboptimal in several regions of the country, with evidence indicating that the magnitude and drivers of HPV vaccine hesitancy vary across geographic settings [31,32,33,34]. In this context, the present study examines parental knowledge, perceived barriers, and hesitancy regarding the HPV vaccine among parents in the Aseer region of Saudi Arabia.

In the present study, parental knowledge regarding HPV infection and HPV vaccination in the Aseer region was limited across several key domains. Gaps were particularly evident in awareness of HPV prevalence, recommended vaccination age, and gender-inclusive vaccination recommendations, with only 47% of parents correctly identifying that the vaccine is recommended for both genders. Although nearly half of parents recognized the association between HPV infection and cancer, a substantial proportion remained uncertain. In contrast, parents expressed generally positive views toward vaccination importance, alongside persistent uncertainty regarding vaccine safety, indicating that favorable attitudes coexist with important knowledge deficits that may affect informed decision-making. These findings are consistent with evidence from Saudi Arabia demonstrating suboptimal parental awareness of HPV and its vaccine. Notably, the only previously published study from the Aseer region similarly reported limited parental knowledge and substantial uncertainty regarding HPV vaccine indications and timing, suggesting that these gaps persist within the same regional population despite increased national attention to HPV vaccination [34]. Comparable knowledge deficiencies have been reported across multiple Saudi regions and populations, indicating that limited awareness of HPV infection and vaccination remains a consistent national challenge [31,32,33,35,36,37]. Collectively, these findings demonstrate persistent knowledge gaps across Saudi regions and populations, reinforcing the relevance of the deficiencies observed among parents in the Aseer region.

Beyond knowledge-related gaps, several structural and perceptual barriers were identified that may further influence parental decisions regarding HPV vaccination. In the present study, concerns related to vaccine safety and insufficient information were the most frequently reported barriers, followed by fear of potential side effects. Consistent with these findings, the previous Aseer-based study identified safety concerns, limited information, and uncertainty as key factors influencing parental acceptance of the HPV vaccine, reinforcing the prominence of belief- and information-based barriers within this community [34]. Similar barriers have been reported in Jeddah and Riyadh, where fear of adverse effects and inadequate information were the most common reasons for vaccine delay or refusal [31,32]. In addition, nearly half of the parents in the present study reported religious or cultural beliefs and the perception that their child was too young to receive the vaccine as barriers. Comparable concerns related to cultural beliefs and misconceptions regarding appropriate vaccination timing have been described in Saudi populations, where moral, religious, and age-related considerations influenced parental decisions [36]. In contrast, financial considerations and perceptions of low infection risk were less commonly reported barriers, suggesting that informational and belief-based factors outweigh access-related issues in shaping parental HPV vaccination decisions. Taken together, these findings suggest that HPV vaccine hesitancy in this population is primarily shaped by perception- and information-related factors rather than structural barriers to access.

In the present study, HPV vaccine hesitancy among parents in the Aseer region showed a balanced distribution, with nearly equal proportions of parents classified as having high hesitancy (48.8%) and low hesitancy (51.2%). This pattern indicates that parental attitudes toward HPV vaccination in Aseer remain divided, reflecting substantial uncertainty and variability in confidence toward HPV vaccination. Compared with national evidence from Saudi Arabia, where approximately one-third of parents were classified as hesitant using a WHO-based Vaccine Hesitancy Scale [22], the proportion of parents demonstrating higher hesitancy in the present study appears comparatively greater. This difference may suggest regional variation in parental perceptions, awareness levels, or sociocultural influences affecting vaccine decision-making. Although national analyses reported geographic differences in hesitancy across Saudi regions, the higher hesitancy observed in Aseer underscores the importance of localized assessments to better understand context-specific determinants of vaccine acceptance. Differences between studies may also reflect variations in study design, measurement approaches, and analytical classification of hesitancy. At the global level, a recent systematic review and meta-analysis estimated that approximately 40% of parents exhibit hesitancy toward HPV vaccination, with safety concerns, limited perceived benefits, and low trust in healthcare providers consistently identified across diverse settings [30].

Analysis of individual hesitancy scale items in the present study revealed a pattern of concurrent confidence and concern among parents. While parents generally expressed favorable beliefs regarding vaccine effectiveness, benefits to child and community health, and willingness to follow healthcare provider recommendations, safety-related concerns, particularly worries about side effects and the relative newness of the vaccine, remained prominent. Similar patterns of favorable vaccine attitudes coexisting with safety concerns have been consistently reported across Saudi Arabia and broader Arab populations, where hesitancy often reflects uncertainty rather than outright refusal [22,28,29]. While the only previously published study from the Aseer region examined parental knowledge, acceptance, and perceived barriers, it did not include a structured assessment of vaccine hesitancy [34]. The present study, therefore, extends the regional literature by providing the first quantitative characterization of HPV vaccine hesitancy in Aseer using a validated WHO-based scale, allowing for more nuanced differentiation between confidence, perceived risk, and trust-related concerns.

Regarding factors associated with hesitancy, lower hesitancy scores were observed among older parents, those with higher educational attainment, parents of fully vaccinated children, and those who expressed trust in healthcare provider recommendations. These findings align with national Saudi evidence indicating that age, education, prior vaccination experience, and provider endorsement were associated with HPV vaccine hesitancy, although the magnitude of these associations varies across populations [22]. This aligns with evidence from the Gulf region indicating that prior positive experiences with routine childhood immunization are associated with greater acceptance of newer or optional vaccines, including HPV [29].

In multivariable regression analysis, older caregiver age and attitude- and trust-related factors, particularly perceived importance of HPV vaccination and willingness to vaccinate following a trusted physician recommendation, remained independently associated with lower hesitancy, whereas knowledge-related variables and child vaccination history were not independently associated after adjustment. In contrast, hesitancy was not significantly associated with child gender, caregiver gender, caregiver relationship to the child, or prior awareness of the HPV vaccine, suggesting that trust-based and experiential factors may be more strongly associated with hesitancy than demographic characteristics alone.

The findings indicate that HPV vaccine hesitancy in the Aseer region appears to be primarily associated with safety concerns, informational gaps, and trust-related factors rather than access barriers. The strong association between lower hesitancy and healthcare provider recommendations highlights the important role of frontline healthcare professionals, including physicians and pharmacists, in addressing parental concerns and supporting informed decision-making regarding HPV vaccination.

## 5. Limitations

This study is limited by its cross-sectional design and reliance on self-reported data, which preclude causal inference and may be subject to recall and social desirability bias. In addition, the regional focus may limit generalizability; however, it provides valuable context-specific insights into HPV vaccine hesitancy in Aseer.

## 6. Conclusions

This study provides the first structured assessment of HPV vaccine hesitancy among parents in the Aseer region. The HPV vaccine hesitancy among parents in the Aseer region was relatively high and mainly associated with caregiver age, educational level, and attitude- and trust-related factors, particularly willingness to vaccinate following a physician recommendation. This highlights the central role of healthcare professionals in supporting HPV vaccine acceptance. Despite generally positive views on the vaccine benefits, important knowledge gaps remain concerning HPV prevalence, recommended vaccination age, and vaccination for both genders. To improve vaccine uptake, targeted education addressing vaccine safety and benefits, active involvement of healthcare providers, culturally tailored outreach strategies, and improved access to vaccination services are recommended.

## Figures and Tables

**Table 1:** Sociodemographic characteristics of parent participants (*n* = 379).

	Category	*n*	%
Age	18–25	95	25.1
	26–35	110	29.0
	36–45	90	23.7
	46+	84	22.2
Gender	Female	205	54.1
	Male	174	45.9
Education Level	High school or less	85	22.4
	Diploma/bachelor’s	210	55.4
	Postgraduate	80	21.1
Heard of vaccine	Yes	320	84.4
	No	59	15.6

**Table 2:** Parental knowledge and awareness of HPV and the HPV vaccine.

Question	Yes *n* (%)	Unsure *n* (%)	No *n* (%)
HPV can cause certain cancers	180 (47.5%)	170 (44.9%)	23 (6.1%)
The HPV vaccine is recommended for both genders	178 (47.0%)	132 (34.8%)	63 (16.6%)
The HPV vaccine is recommended for children between the ages of 9 and 12 years.	142 (37.5%)	229 (60.4%)	8 (2.1%)
The HPV infection is common	112 (29.6%)	135 (35.7%)	132 (34.8%)
It is important for your child to receive the HPV vaccine	209 (55.1%)	122 (32.2%)	41 (10.8%)
The HPV vaccine is safe	187 (49.3%)	153 (40.4%)	30 (7.9%)

**Table 3:** Barriers to HPV vaccination among parents (*n* = 379).

Barrier	YES (*n*)	Percentage (%)
Safety concerns	250	82.6
Not enough information	243	80.3
Fear of side effects	240	79.4
Religious or cultural beliefs	151	49.9
Belief that child is too young	149	49.2
Cost/affordability	111	36.7
My child is not at risk	102	33.7

**Table 4:** Parental attitudes and beliefs toward the HPV vaccine: mean scores on the hesitancy scale (*n* = 379).

No.	Scale Item	Mean (M)	SD
1	I trust the information I have received about the HPV vaccine.	3.53	1.01
2	I believe the HPV vaccine is effective in preventing infection.	3.72	0.97
3	I believe the HPV vaccine will help protect my child’s health.	3.79	0.96
4	I think the HPV vaccine is beneficial for my child.	3.68	0.98
5	I would follow my healthcare provider’s recommendation to vaccinate my child.	3.72	0.97
6	I believe the HPV vaccine is important for my child’s overall health.	3.70	0.91
7	I believe the HPV vaccine is important for the health of the community.	3.75	0.96
8	I am concerned that the HPV vaccine has not been available long enough to know if it is safe.	3.66	1.05
9	I am worried about serious side effects from the HPV vaccine.	3.78	1.08

**Table 5:** Distribution of parents by level of HPV vaccine hesitancy.

Hesitancy Category	*n*	%
Low Hesitancy	194	51.2%
High Hesitancy	185	48.8%

**Table 6:** Differences in parental HPV vaccine hesitancy according to study variables.

Factor	Test Statistic	*p*-Value
Gender of child	t = 0.53	0.594 ^a^
Relationship to child	F = 2.13	0.121 ^b^
Parent’s gender	t = −0.57	0.571 ^a^
Age	F = 3.26	0.022 ^b^
Education level	F = 2.71	0.03 ^b^
Vaccination status	F = 5.67	0.004 ^b^
Heard of vaccine	t = 1.18	0.238 ^a^
Discussion of the HPV vaccine with healthcare provider	t = −1.73	0.084 ^a^
Vaccination of the child if trusted doctor recommends it	t = 5.63	<0.001 ^a^

^a^ independent *t*-test; ^b^ one-way ANOVA.

**Table 7:** Multiple linear regression analysis of factors independently associated with HPV vaccine hesitancy.

Predictor	B	SE	β	t	*p*	95% CI for B	VIF
Constant	48.11	3.16	—	15.24	<0.001	[41.90, 54.32]	—
Parent age	−0.92	0.33	−0.14	−2.77	0.006 **	[−1.58, −0.27]	1.04
Child gender	−1.40	0.77	−0.10	−1.82	0.070	[−2.91, 0.12]	1.13
Highest education level	0.77	0.36	0.11	2.12	0.035 *	[0.06, 1.49]	1.10
Relationship to child	−1.02	0.48	−0.11	−2.12	0.035 *	[−1.97, −0.07]	1.11
HPV can cause cancers	−0.45	0.40	−0.06	−1.13	0.257	[−1.24, 0.33]	1.26
Vaccine recommended for boys & girls	−0.06	0.42	−0.01	−0.13	0.894	[−0.88, 0.77]	1.20
HPV infection is common	0.12	0.50	0.01	0.24	0.813	[−0.87, 1.10]	1.39
Heard about HPV vaccine before	0.05	0.82	0.00	0.06	0.956	[−1.57, 1.67]	1.33
Importance of child receiving HPV vaccine	−1.05	0.43	−0.14	−2.46	0.014 *	[−1.90, −0.21]	1.30
Belief that vaccine is safe	−0.11	0.41	−0.02	−0.27	0.787	[−0.92, 0.70]	1.31
Child received HPV vaccine	−0.22	0.57	−0.02	−0.39	0.697	[−1.35, 0.90]	1.51
Discussed with healthcare provider	−1.06	0.95	−0.07	−1.11	0.266	[−2.94, 0.81]	1.53
More likely if doctor recommends	−5.64	1.22	−0.24	−4.64	<0.001 ***	[−8.03, −3.25]	1.08

* *p* < 0.05; ** *p* < 0.01; *** *p* < 0.001.

## Data Availability

Data are contained within the article.
